# Determinants of undernutrition among children under-five years old in southern Ethiopia: does pregnancy intention matter? A community-based unmatched case-control study

**DOI:** 10.1186/s12887-020-2004-7

**Published:** 2020-03-03

**Authors:** Mohammed Feyisso Shaka, Yetayal Birhanu Woldie, Hirbaye Mokona Lola, Kalkidan Yohannes Olkamo, Adane Tesfaye Anbasse

**Affiliations:** 1 Department of Reproductive Health, Dilla University College of Health Sciences and Medicine, Dilla, Ethiopia; 2 Department of Psychiatry, Dilla University College of Health Sciences and Medicine, Dilla, Ethiopia

**Keywords:** Child malnutrition, Ethiopia, Stunting, Under five, Pregnancy, Unintended

## Abstract

**Background:**

Stunting, which describes a small height for one’s age, is an indicator of chronic malnutrition. It develops mainly as a result of prolonged food deprivation or a chronic disease or illness. Unintended pregnancies and unplanned births are among the psychological factors that negatively affect the nutritional status of children. Therefore, this study aimed to determine the effects of unintended pregnancies and other family and child characteristics on the nutritional status of children under 5 years old.

**Methods:**

A community-based unmatched case-control study was conducted among 302 children (151 cases and 151 controls) 6–59 months old in Wonago town, Gedeo Zone, Southern Ethiopia. The cases were stunted children and the controls were non-stunted children in the study area. The cases were randomly selected from among the stunted children, and the controls were randomly selected from among the non-stunted children. The descriptive characteristics of the respondents were compared using the chi-squared test, and a multivariable logistic regression was used to assess the effects of an unintended pregnancy on stunting, after controlling for the other variables, with a *p* value of 0.05.

**Results:**

The result revealed that unintended pregnancy is found to be among predictors of stunting where children from unintended pregnancy were about three times more likely to be stunted [AOR: 2.62, CI: (1.26, 5.45)]. The other predictors identified in this study were educational status of the father, wealth index of the household and daily meal frequency. From the finding, children from illiterate fathers [AOR: 3.43, CI: (1.04, 11.29)], children from poorer household economic status [AOR: 2.32, CI: (1.20, 4.49)] and children whom their daily meal frequency is below the recommended number of feeding [AOR: 4.50, CI: (1.31, 15.49)] were found to be more stunted.

**Conclusions:**

Based on the results of this study, the children born from unintended pregnancies exhibited a significantly higher risk of stunting. Therefore, preventing unintended pregnancy could play a great role in decreasing the risk of stunting in children.

## Background

Despite the considerable progress made during the past decade in reducing its burden, child undernutrition, which includes conditions such as stunting, underweight, and wasting, contributes to approximately one-half of all childhood mortalities. It is still a major public health problem, particularly in resource-poor countries [1, 2]. Stunting, or a small height for one’s age, describes decelerated or arrested linear growth, and it is an indicator of chronic malnutrition [3]. It mainly develops as a result of prolonged food deprivation, or due to a chronic disease or illness [1].

Regardless of the importance of early childhood nutrition for survival and long-term development, there is no consensus among the global nutrition communities on how to best combat child undernutrition [4, 5]. Tackling the challenge of undernutrition requires cross-sector collaboration, innovative approaches, and optimizing the use of all available interventions [6]. A large number of children under 5 years old die each year from malnutrition-related causes, representing nearly one-half of all the deaths in this age group [2]. Moreover, the survivors also exhibit impaired physical growth and intellectual development, which ultimately reduce their adulthood productivity. On a grander scale, this prevents some nations from achieving their full economic potentials [6].

Globally, an estimated 165 million children under 5 years old (26%) are stunted, 52 million (8%) exhibit wasting, and 100 million (16%) are underweight [7]. Malnutrition contributes to 11% of the disability-adjusted life years (healthy years of life) lost worldwide [8]. In Ethiopia, malnutrition in general, and malnutrition in children under 5 years old in particular, are significant health challenges. Moreover, 44% of the children are stunted, 10% exhibit wasting, and 29% are underweight in Ethiopia [9]. Stunting was estimated to cause a loss of approximately 44 billion Ethiopian Birr from 2005 to 2015 [10].

Although stunting often begins in utero, several research studies have identified socio-demographic, socioeconomic [11–16], environmental [17, 18], dietary [12, 18, 19], parasitic infection and other related illness [11, 20], and psychological [19] correlations with stunting. One of the psychological factors that might be associated with children stunting is unintended pregnancy, either unwanted (the parent did not desire any or any more children) or mistimed (the pregnancy occurred earlier than desired) [16, 21, 22]. Evidence has suggested that family planning (FP), a proxy indicator of one’s pregnancy intention, can have a significant influence on achieving key nutrition outcomes. Previous research has shown that when planned pregnancies occur, the feeding practices (including breastfeeding) are improved, resulting in an improvement in the nutritional outcomes of the children [6]. There is also ample evidence of the link between unwanted pregnancies and adverse neonatal outcomes and behaviors, including a low birth weight, neonatal mortality, the absence of breast-feeding, and poor parental care [23, 24]. A study conducted by Marston and Cleland in Peru found a 15% greater risk of stunting among children from unwanted pregnancies when compared to those from wanted pregnancies. However, the findings of a study from Egypt were contrary, in that the likelihood of stunting was lower if a pregnancy was mistimed or unwanted than if it was wanted [24]. Barber and colleagues hypothesized that various pathways linked unwanted childbearing, child health, and mother-child relationships. They suggested that children who were unwanted at the time of conception may face more neglect and abuse than those who were wanted [25].

Even though there has been a trend toward a drop in the global pregnancy rates, the proportion of unintended pregnancies remains high, particularly in the developing world [6]. During 2012, approximately 40% of the pregnancies worldwide (or 85 million pregnancies) were estimated to be unintended. Moreover, studies conducted in different regions of Ethiopia revealed unintended pregnancy rates ranging between 27.9 and 42.4% [26–28], and nearly one million unintended pregnancies are expected to occur each year in Ethiopia [29]. Unintended pregnancies are of concern from both a human rights perspective and a public health perspective, and the consequences of unintended pregnancies are serious. They impose considerable burdens on children, women, men, and families [30].

Such high rates of unintended pregnancy and its possible series repercussion justify a study of their negative consequences on child nutrition and growth, especially when considering that adverse nutritional outcomes in a child are more likely to occur if the pregnancy was unintended. In addition, the study area is one of the areas with the highest burden of malnutrition [31] and very poor family planning utilization in the country [32]. This study hypothesized that high number of unintended pregnancies that could be resulted from poor family planning in the study area might be contributed for such significant burden of under-five malnutrition Therefore, the study was designed to assess the association of unintended pregnancies and other family and child characteristics with the nutritional status of children, particularly with regard to stunting among children under 5 years old. There are no studies showing such side of the factors contributing for the burden of malnutrition.

## Methods

### Study design and setting

A community-based unmatched case-control study was conducted among children from 6 to 59 months of age residing in Wonago town, Gedeo Zone, SNNPR, Ethiopia. This town is located 370 km from Addis Ababa (the capital of Ethiopia) and 12 km from Dilla town (Gedeo Zone administrative center). The town is the administrative center of Wonago rural wereda. The town has a longitude of 382,667 (3816′0.120″ E) and latitude of 63,167 (619′ 0.012″ N) with altitude of 1776. The dominant ethnic group in the area is Gedeo and most of the people speak Gedeo-Offa language [31]. The town has two sub-divisions. This study was conducted from June 01–25, 2017.

### Population

In this study, cases (stunted children) were children with height/length –for- age z-score less than − 2 SD and controls (non-stunted children) were children having height/length –for- age z-score greater than or equal to − 2 SD according to WHO standards. Cases were randomly selected stunted children from among all stunted children of 6–59 months in the study area and controls were randomly selected non-stunted children among all non-stunted children of 6–59 months in the study area. Children with known underlying chronic illnesses and congenital or chromosomal abnormalities were excluded from the study.

### Sample size determination and sampling procedures

The sample size was determined using two population proportion formula by the Epi-Info 7.1 software, with the following assumptions:- *p* = 28%, which estimates the proportion of non-stunted children who are the outcome of an unintended pregnancy, **α** = 1.96 critical value at 95% confidence interval of certainty**,** OR = 2.1, from literature review, stunted children were expected to come from unintended pregnancy when compared to non-stunted children, power of 80%**,** r = 1 which is, the ratio of cases to controls 1:1. Using the above assumptions, the sample size became 302, meaning is 151 for cases and 151 for controls.

With regard to the sampling procedure, primarily, a survey was conducted to identify the number of children under the age of five within the study area, and their respective nutritional status. The survey was carried out by trained health professionals who have previous similar experience on anthropometric measurement. They enumerated the children, coded them and measured height and weight of the children. Based on the nutritional status index derived from the height and weight measured during the survey, the total children in the study area were labeled into two groups (stunted and non-stunted) and each children were coded. Using simple random sampling method, 151 stunted children (cases) were selected from among all stunted children and 151 non-stunted children (controls) were selected from among all non-stunted children identified during the survey. This was done using table of random numbers for both groups (stunted and non-stunted children) separately. Fortunately, all of the respondents identified by simple random sampling from both group were successfully responded for the enquiry.

### Data collection instruments, personnel and quality assurance

The questionnaire was adapted from Ethiopian health and demographic survey [32] questionnaire employed for assessment of child malnutrition. It was further developed using peer reviewed published literatures to include determinants of malnutrition, including unintended pregnancy. These consists of socio-economic, socio-demographic, child characteristics, child caring practices, pregnancy intention and environmental health condition/sanitation. The questionnaire was further modified after a pretest was conducted. Finally, the tool was translated into local language for field work purposes and back to English for checking language consistency.

Weight was measured to the nearest 0.1 digits in kilograms with minimum clothing and no shoes. The salter spring scale with the capacity of measuring 25 Kg was used for younger children and the battery powered digital scale (SECA, UNICEF, Copenhagen) was used for older children. Weighing scales were calibrated with known weight objects regularly. The ace of scale indicator was checked against a zero reading after weighing every child. Height and length were measured using a standardized measuring board to the nearest 0.1 cm. Fourteen data collectors, who are diploma nurses, were recruited from the study area and priority was given to those with previous experience with data collection. Two supervisors were assigned to control the overall field work. The data collection was done through face-to face interview by trained data collectors for all participants (parents of the selected children).

### Measurements

The weight and height of the children was converted into weight-for-age and height-for-age standard deviation units (z-scores) using ENASMART software based on WHO Child Growth Standards. The children were classified as stunted if their height for age z-score was below − 2. Maternal pregnancy intention was determined by asking women to retrospectively recall their feelings at the time of conception for each birth within the past five years. Women were asked whether the pregnancy had been planned (wanted at that time), mistimed (wanted later), or unwanted (not wanted at any time). To measure pregnancy intention, the respondents were asked: “At the time you became pregnant with [name of last-born child], did you want to become pregnant then, did you want to wait until later, or did you want no more children at all?” Intended pregnancies were defined as those pregnancies to mothers who wanted the pregnancy at that time. Unwanted pregnancies were those to mothers who did not want to have more children; and mistimed pregnancies referred to mothers who wanted to become pregnant eventually, but at a later time. The questions and definitions used here are standard for reproductive health surveys conducted worldwide.

Underweight was defined as children with weight for age z-score less than − 2 and wasting was defined as weight for height z-score less than − 2. For measurement of deworming status, children who got age specific dose of albendazole or mebendazole within the last six months according to deworming protocol in Ethiopia.

Meal frequency was classified as adequate or inadequate based on the age specific number of meal frequency recommendation taken from WHO guideline of indicators for assessing infant and young child feeding practices [33] and OCHA indicators registry for older children [34].

### Data analysis

Data entry was done using EpiData version 3.1 by single data entry method and exported to statistical package for social sciences (SPSS) version 20 for analysis. Descriptive statistics were computed for nutritional status, pregnancy intention, socio-demographic and socio-economic characteristics. The proportion of unintended pregnancy and other important variables were compared among case children and control children using chi-square test. Assessment of crude association between stunting and each independent variables at a time was conducted using univariate logistic regression analysis.

Multivariable logistic regression was used to assess the effect of independent variables including the pregnancy intention on the outcome variable (stunting). Crude and adjusted odds ratios with their corresponding 95% confidence intervals were computed. A *p*-value ≤0.05 were considered statistically significant in this study. Efforts were made to assess the fulfillment of the necessary assumptions for the application of multiple logistic regression. In this regard, the Hosmer and Lemeshow’s goodness-of-fit test was done to check the model fit. Interaction between different predictor variables was checked using variable inflation factor (VIF). Particularly interaction between the educational level of family and household wealth was checked.

## Result

### Socio-demographic and socio-economic characteristics of the child and mother

The study participants were 151 cases of stunted children and 151 controls (non-stunted children). Among these participants, 105 (70%) of the cases and 85 (56.3%) of the controls were male children. The age distribution of participants showed that about 97 (65%) of the cases and 102 (63.5%) of the control children were 2 years or above. The mean age of the mother/caregiver of the participants was 29.8 (±5.8 SD) years for cases and 29.0 (±5.9 SD) years for controls. About 93 (61.5%) of the mothers of case children and 67 (45.9%) of the mothers of control children were illiterate. Among the socio-demographic characteristics, sex of the index child (*p* = 0.017), mother’s education (*p* = 0.002), and father’s education (*p* = 0.005) were significantly associated with stunting, which was found by bivariate analysis (Table 1).

**Table 1:** Univariate logistic regression analysis of socio-demographic and socio-economic characteristics of the child and mother in Wonago town, Gedeo Zone, South Ethiopia, June 2017

No.	Variables	Cases	Controls	COR (95% CI)	*P*-value
1.1.1.1	Age of the child	151	151		0.53
	6–24 months	54 (35.7)	49 (32.5)	1.04 (0.62,1.74)	
	25 to 36 months	27 (17.9)	36 (23.8)	0.71 (0.39,1.29)	
	above 36 months	70 (46.4)	66 (43.7)	1	
	Mean age in months (SD)	33.6 (15.6)	33.8 (15.1)	–	–
1.1.2	Sex	151	151		0.017
	Male	105 (69.5)	85 (56.3)	1	
	Female	46 (30.5)	66 (43.7)	0.56 (0.35,0.90)	
1.1.1.3	Mother age	151	151		0.160
	less than 25 years	22 (14.6)	32 (21.2)	0.50 (0.25,1.02)	
	25 to 34 years	84 (55.6)	86 (57.0)	0.72 (0.42,1.23)	
	35 years and above	45 (29.8)	33 (21.9)	1	
	Mean age in years (SD)	29.8 (5.8)	29.0 (5.9)	–	–
1.1.1.1.4	Mother occupation	151	151		0.23
	Housewife	75 (49.7)	74 (49.0)	1	
	Merchant	39 (25.8)	50 (33.1)	0.77 (0.45,1.30)	
	Farmer	11 (7.3)	12 (7.9)	0.90 (0.38,2.18)	
	Other	26 (19.2)	15 (9.9)	1.71 (0.84,3.49)	
1.1.1.1.5	Father occupation	151	145		0.123
	Farmer	61 (40.4)	43 (29.7)	1	
	Merchant	41 (27.2)	57 (39.3)	0.51 (0.29,0.89)	
	Daily laborer	25 (16.6)	23 (15.9)	0.77 (0.38,1.52)	
	Other	24 (15.9)	22 (15.2)	0.77 (0.38,1.54)	
1.1.1.6	Mother education	151	146		0.002
	Illiterate	93 (61.5)	67 (45.9)	4.63 (1.76,12.14)	
	Primary(1–8)	52 (34.4)	59 (40.4)	2.94 (1.10,7.87)	
	Secondary and above	6 (4.0)	20 (13.7)	1	
1.1.1.7	Father education	151	145		0.005
	Illiterate	40 (26.5)	19 (13.1)	3.39 (1.59,7.20)	
	Primary(1–8)	88 (58.3)	89 (61.4)	1.59 (0.87,2.89)	
	Secondary and above	23 (15.2)	37 (25.5)	1	
1.1.8	Head of the HH	145	144		0.193
	Husband	102 (67.5)	111 (77.1)	1	
	Wife	43 (28.5)	33 (22.9)	1.42 (0.84,2.40)	
1.1.9	Family size	151	148		0.552
	4 or less	53 (35.1)	61 (41.2)	1	
	5 to 8	82 (54.3)	73 (49.3)	1.29 (0.80,2.10)	
	above 8	16 (10.6)	14 (9.5)	1.31 (0.59,2.95)	
10	Number of siblings	151	148		0.406
	Three or less	72 (47.7)	82 (55.4)	1	
	Four to six	63 (41.7)	52 (35.1)	1.38 (0.85, 2.24)	
	Above six	16 (10.6)	14 (9.5)	1.30 (0.59, 2.85)	
1.1.11	Wealth index	148	149		0.009
	Low	15 (10.1)	27 (18.1)	0.76 (0.36,1.62)	
	Middle	95 (64.2)	70 (47.0)	1.86 (1.10,3.12)*	
	High	38 (25.7)	52 (34.9)	1	
1.1.12	Daily food need of family	150	144		0.088
	Produce their own	50 (33.3)	35 (24.3)	1	
	Purchase	100 (66.7)	109 (75.7)	0.64 (0.39,1.07)	

From the total children who participated in this study, 95 (64.2%) of case children and 70 (47.0%) of the control children were from households belonging to middle wealth index. About two-third of the case children family and three-fourth of the control children family achieved daily food need of the household through purchase from the market. On bivariate analysis, wealth index of the household was found to affect under five children stunting (*p* = 0.009) (Table 1).

### Maternal reproductive health and child feeding related information of respondents

Among all children, 41 (27.2%) of the case children and 26 (17.2%) of the control children were delivered from unintended pregnancy and this difference was statistically significant with *p*-value of 0.038. A significant difference was not observed among cases and controls taking in to account the place of delivery of the index child (p-value = 0.221). Regarding decision making power of the mothers in the household, 109 (72.2%) mothers of the case children and 101 (67.3%) of the mothers of the control children had no decision making power. The bivariate analysis result showed that pregnancy status (*p* = 0.038) and decision making power of the mother in the household (*p* = 0.010) significantly affected stunting (Table 2).

**Table 2:** Univariate logistic regression analysis of maternal reproductive health and child feeding related information of respondents in Wonago town, Gedeo Zone, South Ethiopia, June 2017

No.	Variables	Cases	Controls	COR (95%CI)	*P*-value
1.1.1.1	Age at first birth	151	151		0.621
	below 18	48 (31.8)	41 (27.2)	1.12 (0.55,2.30)	
	18–24	80 (53.0)	88 (58.3)	0.87 (0.45,1.68)	
	25 and above	23 (15.2)	22 (14.6)	1	
1.1.1.1.2	Birth order	151	150		0.28
	1st	43 (28.5)	47 (31.3)	0.66 (0.37,1.17)	
	2nd	25 (16.5)	31 (20.7)	0.58 (0.30,1.13)	
	3rd	26 (17.2)	31 (20.7)	0.60 (0.31,1.16)	
	4th and above	57 (37.7)	41 (27.3)	1	
1.1.3	Pregnancy status	151	151		0.038
	Intended	110 (72.8)	125 (82.8)	1	
	Unintended	41 (27.2)	26 (17.2)	1.79 (1.03,3.12)	
1.1.4	ANC *visit	149	148		0.299
	Yes	108 (72.5)	115 (77.7)	1	
	No	41 (27.5)	33 (22.3)	1.32 (0.78,2.24)	
1.1.5	Place of delivery	148	147		0.221
	Home	87 (58.8)	76 (51.7)	1	
	Health institution	61 (41.2)	71 (48.3)	0.75 (0.47,1.19)	
1.1.6	Post natal care	135	140		0.217
	Yes	84 (62.2)	97 (69.3)	1	
	No	51 (37.8)	43 (30.7)	1.37 (0.83,2.26)	
1.1.1.7	Decision making power	151	150		0.010
	no decision power	109 (72.2)	101 (67.3)	0.87 (0.50,1.52)	
	less decision power	5 (3.3)	19 (12.7)	0.21 (0.07,0.64)	
	fair decision power	37 (24.5)	30 (20.0)	1	
1.1.1.8	Length of breast feeding	150	145		0.470
	inadequate	17 (11.3)	19 (13.1)	0.99 (0.47,2.11)	
	Adequate	79 (52.7)	66 (45.5)	1.33 (0.81,2.17)	
	Excess	54 (36.0)	60 (41.4)	1	
1.1.9	Minimum meal frequency	149	150		0.010
	Adequate	125 (83.9)	144 (96.0)	1	
	Not adequate	24 (16.1)	6 (4.0)	4.61 (1.83,11.63)	
1.1.10	Dewarming	144	140		0.68
	Yes	113 (78.5)	107 (76.4)	1	
	No	31 (21.5)	33 (23.6)	0.89 (0.51,1.55)	

*Antenatal care

On the other hand, almost all of the children from the case children (147; 99.3%) and those from the control children (139; 98.6%) had breast fed or were breast feeding during the time of data collection. The majority of the case children (133; 93.0%) and control children (137; 92.6%) had begun complementary feeding at the recommended age. One hundred and twenty-five (83.9%) of the case children and 144(96.0%) of the control children had an adequate daily meal frequency. Nutritionally, 104 (68.9%) of the case children and 37 (24.5) the control children were underweight, while 36 (23.8%) of the case children and 21(13.9%) of the control children were wasting.

Among the children who participated in the study, 113 (78.5%) of the case children and 107(76.4%) of the control children had been dewormed within the previous six months. The minimum daily meal frequency of the children was significantly associated with stunting among children under five upon bivariate analysis (*p*-value 0.010) (Table 2).

### Determinants of malnutrition among children under five

Multivariable logistic regression was used to assess the effect of unintended pregnancy on the nutritional status of children in this study. The results revealed that children from unintended pregnancies were 2.6 times more likely to be stunted than children from intended pregnancies [AOR: 2.62, CI: (1.26, 5.45)]. The other predictors identified in this study were the educational status of the father, the wealth index of the household, and the child’s daily meal frequency (the frequency of complementary feeding for breast-feeding or the usual meal per day for the non-breast feeding scenario). Children whose fathers were illiterate had more than thrice as high risk of stunting than those whose fathers attended secondary school or attained a higher educational level [AOR: 3.43, CI: (1.04,11.29)].

Regarding the wealth status of the family, children of a middle-level wealth status had a more than twice as high risk of stunting [AOR: 2.32, CI: (1.20, 4.49)] than those of a higher-level wealth status. Similarly, children whose daily meal frequencies were inadequate (below the daily recommended frequency) were 4.5 times more likely to be stunted [AOR: 4.50, CI: (1.31, 15.49)] (Table 3).

**Table 3:** Determinants of malnutrition among under five children in Wonago town, Gedeo Zone, South Ethiopia, June 2017- results of multivariate logistic regression analysis

No.	Variables	Cases	Controls	COR (95% CI)	*AOR (95% CI)
1.1.1	Sex	151	151		
	Male	105 (69.5)	85 (56.3)	1	1
	Female	46 (30.5)	66 (43.7)	0.56 (0.35,0.90)	0.72 (0.40,1.32)
1.1.1.2	Mother age	151	151		
	less than 25 years	22 (14.6)	32 (21.2)	0.50 (0.25,1.02)	0.58 (0.22,1.53)
	25 to 34 years	84 (55.6)	86 (57.0)	0.72 (0.42,1.23)	1.07 (0.52,2.23)
	35 years and above	45 (29.8)	33 (21.9)	1	1
1.1.1.1.3	Father occupation	151	145		
	Farmer	61 (40.4)	43 (29.7)	1	1
	Merchant	41 (27.2)	57 (39.3)	0.51 (0.29,0.89)	0.54 (0.23,1.28)
	Daily laborer	25 (16.6)	23 (15.9)	0.77 (0.38,1.52)	0.47 (0.19,1.18)
	Other	24 (15.9)	22 (15.2)	0.77 (0.38,1.54)	2.05 (0.67,6.27)
4	Mother education	151	146		
	Illiterate	93 (61.5)	67 (45.9)	4.63 (1.76,12.14)	1.50 (0.38,5.95)
	Primary(1–8)	52 (34.4)	59 (40.4)	2.94 (1.10,7.87)	1.60 (0.43,5.95)
	Secondary and above	6 (4.0)	20 (13.7)	1	1
1.1.1.5	Father education	151	145		
	Illiterate	40 (26.5)	19 (13.1)	3.39 (1.59,7.20)	**3.43 (1.04,11.29)**
	Primary(1–8)	88 (58.3)	89 (61.4)	1.59 (0.87,2.89)	1.51 (0.58,3.97)
	Secondary and above	23 (15.2)	37 (25.5)	1	1
1.1.1.6	Wealth index	148	149		
	Low	15 (10.1)	27 (18.1)	0.76 (0.36,1.62)	1.10 (0.41,2.97)
	Middle	95 (64.2)	70 (47.0)	1.86 (1.10,3.12)	**2.32 (1.20,4.49)**
	High	38 (25.7)	52 (34.9)	1	1
1.1.7	Source of food for family	150	144		
	Produce their own	50 (33.3)	35 (24.3)	1	1
	Purchase	100 (66.7)	109 (75.7)	0.64 (0.39,1.07)	1.76 (0.77,4.02)
1.1.8	Minimum meal frequency	149	150		
	Adequate	125 (83.9)	144 (96.0)	1	1
	Not adequate	24 (16.1)	6 (4.0)	4.61 (1.83,11.63)	**4.50 (1.31,15.49)**
1.1.9	Postnatal care visit	135	140		
	Yes	84 (62.2)	97 (69.3)	1	1
	No	51 (37.8)	43 (30.7)	1.37 (0.83,2.26)	0.85 (0.45,1.60)
1.1.10	Pregnancy status	151	151		
	Intended	110 (72.8)	125 (82.8)	1	1
	Unintended	41 (27.2)	26 (17.2)	1.79 (1.03,3.12)	**2.62 (1.26,5.45)**
1.1.1.11	Decision power	151	150		
	no decision power	109 (72.2)	101 (67.3)	0.87 (0.50,1.52)	1.15 (0.53,2.53)
	less decision power	5 (3.3)	19 (12.7)	0.21 (0.07,0.64)	0.40 (0.11,1.46)
	fair decision power	37 (24.5)	30 (20.0)	1	1

*C*ontrolled for all variables included in the table,*

*AOR Adjusted Odds Ratio, CI Confidence Interval, COR Crude Odds Ratio*

## Discussion

Apart from the usual predictors of nutritional status of children, reproductive health related factors particularly, pregnancy intention, was identified to significantly affect stunting among children. In this study, children whose conception had been unintended were significantly at greater risk of stunting than children whose conception was intended. Accordingly, children from unintended pregnancies were about three fold more likely to be stunted compared to those from intended pregnancies. This finding is also supported by finding from Bolivia [16], and finding from Bangladesh Demographic and Health Survey [22] which reported higher proportion of stunting among children born from unintended pregnancies. This might be due to the fact that, children born from unintended pregnancies were more likely to be exposed to conscious or unconscious neglect, resulting in inappropriate feeding, poor child-mother bond and poor health care seeking for the child. In addition, there might be poor economical and psychological preparation/capability for rearing and feeding of the child, which could substantially affect nutritional status of the child.

The household wealth of the family of the subject children was associated with stunted growth in this study. This finding is in line with other findings from analysis of the Mini Ethiopian Demographic and Health Survey data [15], a cross sectional study conducted on prevalence and determinants malnutrition in Bangladesh [13], and another study from Sindh, Pakistan [14]. The primary influence of household wealth is on child feeding practices related to an inadequate amount of food and the nutritional content of meals, which affect the nutritional status of children. This implies that economic empowerment for households in developing countries may be still an issue that need due attention.

Among the socio-economic factors, the educational status of the father of children was found to be correlated with stunting among children. Children of illiterate fathers were at more than three times higher risk of stunting compared to educated fathers. This finding is also in line with the findings of the study from Bangladesh [13] and other studies [32–36]. Possibly, this might be due to the fact that households with educated fathers have a greater likelihood of having a higher income, since fathers are the main source of income for the households studied. This may also indicate educated fathers have a higher awareness and are more conscience of the nutritional needs of children. Other studies in other locations [11, 15] found that the educational status of the mother was associated with stunting. This was not the case in our study. This might be happened as a result of chance, otherwise we couldn’t find possible reason from the characteristics of the study population.

The other variable found as a predictor for stunting in this study was minimum meal frequency. Children who hadn’t received the recommended daily meal frequency were 4.5 times more likely to be stunted. This finding is also consistent with the result of a study from Burkina Faso [37]. This finding could be explained by the reality that if the child was not getting the complementary food (for breast-feeding children) or regular daily meals (for non-breast-feeding children) as recommended, the possibility of getting adequate nutrient intake will be diminished, which may significantly affect the child’s nutritional status. However, other studies, including those in other areas of Ethiopia, found no association between a child’s daily meal frequency and stunting [12, 38]. This could be due to the difference in the study population and period across the study areas and/or due to the difference in methods of assessment of feeding pattern between the studies.

This study tried to assess the effect of numerous variables, including reproductive health-related information, on stunting among children. As it is a community-based study, cases and controls were selected randomly from the community to address the issue of representation. Though the study has valuable strength, we admit certain important limitations. The main limitation of this study was that while assessing pregnancy intention, we only used information from the mother and didn’t use paternal data. Another limitation is that pregnancy intention was assessed based on retrospective recall of the pregnancy’s status, which could affect the mother’s response. The other considerable limitations of this study are its small sample size, single center (study area), and exclusion of some important variables like dietary diversity.

## Conclusion

Untended pregnancy was found to be an important predictor of stunting in this study, where children from untended conception were at greater risk of stunting. Considerable number of stunted children were from untended pregnancy. Similarly the educational status of the father, the households’ wealth index and the daily meal frequency were significantly associated with children stunting. Children from a poorer households, children whose fathers were illiterate and children with inadequate minimum meal frequency were at greater risk of being stunted. Hence, preventing unintended pregnancy could be a valuable intervention modality for the prevention of stunting among children. Further studies to determine the mechanism through which unintended pregnancies could contribute for child malnutrition should be considered.

## Data Availability

The data underlying this study is readily available and can be accessed as needed from the corresponding author.

## Abbreviations

ANC: Antenatal Care
AOR: Adjusted Odds Ratio
CF: Complementary Feeding
CI: Confidence Interval
COR: Crude Odds Ratio
EDHS: Ethiopia Demographic and Health Survey
HAZ: Height for Age Z-score
MCM: Modern Contraceptive Methods
OCHA: United Nations Office for the Coordination of Humanitarian Affairs
SD: Standard Deviation
WAZ: Weight for Age Z-score
WHZ: Weight for Height Z-score
